# Indents

**Published:** 1903-09-15

**Authors:** 


### INDENTS.

☞ **Brief Articles of Technical or Scientific Value will receive notice and comment. Contributions invited.**

**Lost Voice after Tooth Extraction.** A man in Jersey City had a tooth extracted June 4th, and has not spoken a word since.—*Digest.*

**To Clean Vulcanite Files.** Apply chloroform and clean with stiff brush. This will remove all the rubber packed in trimming plates.—W. H. Wheat, *Dental Hints.*

**Detroit Dental College Alumni Association.** Met June 11th, 1903, and elected the following officers: President, T. J. Collins; Sec'y, J. S. Hall; Treas., E. Frumveller.

**Re-soldering.** To prevent the unsoldering or re-fusing of parts previously united, coat such surfaces with crocus (ferric peroxid), or a solution of plumbago or whiting in alcohol or water.—H. J. Goslee, *Items.*

**To Fasten Teeth to Wax Base Plate.** Warm the tooth and melt on to the pin surface a little adhesive wax, place in position in the arch and fill around it to contour with base-plate wax.—W. H. Taggart, in *Review.*

**Finishing Rubber Plates.** A very glossy surface can be given vulcanite work by using a dry and moderately stiff brush-wheel charged with talcum powder after having finished with pumice or emery.—Dr. F. A. Weld, in *Review.*

**Bicarbonate of of Soda for Toothache.** Duckworth recommends this remedy to allay odontalgia and other painful diseases of the mucous membrane. It seems to act by producing an alkalinity of the blood.—*Merck's Annual.*

**Formaldehyde. Antidote.** In case of poisoning from formaldehyde we have an accessible and reliable remedy in ammonia. It may be given in the form of ammonia water, a few drops well diluted, or the aromatic spirits, or a solution ammonium acetate.—*Merck's Archives.*

**Annealing.** The late Sir W. C. Roberts-Austen defined annealing "as the release of strain in metals which may itself have been produced by mechanical treatment, such as hammering, rolling or wire-drawing, or by either rapid or slow cooling from a more or less elevated temperature."—*Pop. Mechs.*

**Chemical Test for Sodium Dioxid.** Place about 1 gm. of sodium dioxid in a clean, dry test-tube and add one or two c.c. of water. If the chemical is efficient enough oxygen will be generated to inflame a glowing splinter held at the mouth of the tube.

—J. P. Buckley, *Review.*

**Local Anesthesia.** Moller suggests dissolving in eighty drops of a one percent solution of supra-renal extract one twentieth of a grain of hydrochlorate of tropococaine, and one thirtieth of a grain of chloride of sodium. Inject from 30 to 50 drops into the maxillary periosteum.—*Merck's Annual.*

**Guaiocol in Pulpitis.** Holland recommends guaiocol for irritable pulps, and as an addition to arsenical nerve pastes to prevent pains of the cauterant arsenic.—*Merck's Annual.* This remedy is undoubtedly a good anodyne remedy and has good obtundent qualities, but has such an unpleasant odor that it can't be kept in a dental office.—ED.

**To Prevent Formation of Rust in Hypodermic Needle Points** Put the needles in a bottle, cover with pure gasoline, and cork tightly. When wanted for use, blow through the needle and dip in alcohol, and the odor will disappear.—H. L. Pratt, *Dental World.* Don't put your hypodermic needle in the mouth to blow through it, but use the hot-air syringe.—EDITOR.

**Conduct of Dental Colleges.** If honesty could be placed before money interest in the conduct of dental colleges for but a single year, and reasonable standards of admission and study maintained, the commercial aspect of dental education would be properly cared for, and the financial interests of all concerned would very soon be much improved. —Dr. Chittenden, in *American Medical Journal.*

**Neuralgia.** Naegeli announces that he has frequently caused almost immediate cessation of cephalalgia and facial neuralgia, as well as forms of long-continued odontalgia, by simply elevating the larynx, holding it well upward for sixty or seventy seconds. This frequently requires to be repeated several times, but quite as frequently one single attempt will prove succesful—*Public Health Journal.*

**Eugenol for Sensitive Dentine.** Witzel recommends Eugenol as an antiseptic obtundent of dentine. It diffuses both live and dead dentine and has no corrosive action. It is also a good remedy in pulpitis. Dry the cavity well before applying. With formaldehyde it makes a good disinfectant of putrid canals. It is also an anodyne treatment after extracting.—*Merck's Annual.*

**Drainage of Cavities Containing Thick Pus.** Cavities containing thick pus should not as a rule, be drained with gauze, for the the reason that the pus is too thick to be amenable to the laws of capillarity, and because the gauze may also have a tendency to block and practically cork up the wound. Tubes, rolls of protective tissue, silk-worm-gut, and horsehair are to be preferred.—*Internat. Journ. of Surgery.*

**Cleaning the Cement Slab.** I notice from the last "Journal" that some dentists have trouble with the cement slab. I do not. After the "mix" is used, drop the slab in water (dilute ammonia water.—ED. REGISTER),

In working cement with plugger or broaches, dip in water and wipe off occasionally. Cement does not stick to wet instruments—a good thing to know, especially in root filling. —P. H. Callahan in *Texas Journal.*

**Osmic Acid for Exposed Pulps.** In irritations of the pulp Goldberg uses in place of formaldehyde, a three percent ether solution of osmic acid which he applies on cotton sealing to place with a sandarac plug covered with temporary gutta-percha stopping. After twenty-four hours the dressings are removed and the pulp capped with chloropercha and filled with cement.—*Merck's Annual.*

**Celluloid Lacquer.** Dissolve uncolored celluloid in a mixture of strong alcohol and ether. The celluloid first swells up in the solvent, and after vigorous shaking the bottle is allowed to stand quietly for the undissolved portion to settle, when the clear, supernatant fluid is poured off. The latter may be immediately used; it yields a colorless, glossy lacquer, or may be colored, as desired, with aniline colors.

**Thumb-sucking Case.** Dr. Heckard cites a case of distorted arch in a child four years of age, which he cured by having the child blow a horn for five minutes three times each day for five months. At the end of which time the arch had resumed a normal outline, and had not reverted after four months although the horn had not been used during the last four months. It also broke off the thumb-sucking habit.—*Review.*

**Fruit Preserver.** The fumigation of citrus fruits with a preparation of formaldehyde is an experiment that has resulted so successfully as a perservative that California fruit shippers are most enthusiastic over the results obtained. Nearly every box of fruit upon which the experiment was tried in Pasadena recently reached its destination in the East in an almost perfect state of preservation, there being only about 5 percent decay as against a usual loss of about 25 percent.

**Broader Culture.** The laity have not much respect for us in many ways, because we do not show ourselves as worthy of it as we should. Just in proportion as you clothe yourselves with and garner collateral science to decorate and enlarge the field of your profession will you raise its standard. Therefore, I like to see the dentists literary men. I like to see them in other studies than that of dentistry alone, and by doing that I think we will do a great deal.—Dr. Hungerford, in *Western Journal.*

**Protecting Cement Fillings from Moisture.** Mistakes are frequently made by conscientious operators in keeping cement fillings dry too long after insertion. With many of the best oxyphosphates in use to-day the only effective means of preventing shrinkage of the filling is to allow it to become moist shortly after insertion. Manufacturers should test their cements and indicate on the packages the proper length of time after mixing to allow moisture to come in contact with the filling.—*Dental Review.*

**Roughened Base on Porcelain Inlays.** Place an extremely thin layer of very coarse, high-fusing body over the floor and nearly to the margins of the matrix. This spread must be of much higher-fusing porcelain than contained in the body of the inlay. Bake to a *slight* bisque. If the other porcelains are handled correctly the result will be a nicely-granulated surface on the base, which is especially applicable to small and shallow cervical restorations, where the staying qualities are difficult to obtain.—Dr. J. S. Bridges, in *Review.*

**Bridge-work a Compromise.** It is an indisputable fact that all bridge-work is against nature, and we should therefore not be surprised when there are compromises on the part of nature under the circumstances under which she labors. Bridge-work is in many respects the ideal compromise of the profession with untoward existing conditions, and I heartily approve of it, but when you put four teeth on a foundation which nature used for two, you have overloaded her and must not hope for ideal results.—Dr. Stubblefield, in *Digest*.

**Treatment of Congested Gums.** In the treatment of congested gums, whether involved in pyorrhea alveolaris or not, iodide of zinc in the crystal form has often been recommended and applied with much success. It is one of the most effective agents to restore the gums to their normal form, but it occasionally seems to induce sensitiveness of the teeth, and the taste is objectionable to some patients. To overcome these limitations the crystals may be mixed with one of the essential oils to the consistency of a syrup. Applied in this form, it is fully as effective and much more acceptable.—*Dental Review*.

**Listerine as a Preservative of Hydrogen Dioxid.** A mixture of an equal quantity of listerine with peroxide of hydrogen proves effective in maintaining the stability of the peroxide a much greater length of time than would otherwise obtain, and that mouth-washes of alkaline reaction should never be used in combination with peroxide, the alkalinity not only altering its chemical, but to a great extent destroying its antiseptic effect. At best, alkalies simply temporarily neutralize acidity, whereas, a true antiseptic overcomes the conditions to which the acid-forming ferment is due.

**Septoforma.** This is a new disinfectant by Merck. It is made by the condensation products of formic aldehyde and the terpene, naphthaline and phenol groups. These are mixed with alcoholic lanolin soap so as to form a yellowish brown solution, which with water yields a lather. It has powerful bactericidal properties. One and one-half percent solutions are suitable for irrigating purposes. Fresh wounds may be sterilized with three percent and infected wounds with ten percent alcoholic solution. Five percent solutions serve as deodorizers, and for sterilizing instruments. These statements will indicate its use in oral treatments.

**Equivocal Comment.** Professor Andrews, of Edinburgh, entered his class room one morning before his students had assembled and wrote on the blackboard the following announcement:

"Professor Andrews begs to announce to his students that he has been appointed physician to the Queen."

When the first student arrived in the class room, he promptly wrote under the announcement, "God save the Queen!"

Let us then write under the record of each day's work, "God save our patients."—Dr. McFarlane in *Review.*

**Probert at it Again.** Our readers will remember that on several occasions in the past we have exposed the schemes of A. C. Probert, who was the promoter of "the St. Luke's Hospital," at Niles, Mich. We warned the profession against buying any of the bogus certificates, medals, etc., which Probert offered, so he gave up the scheme. He has recently been arrested on complaint of Dr. John B. Murphy, the celebrated surgeon, for fraudulently using Dr. Murphy's name in promoting "the Christian Hospital," an institution which sold certificates, etc., to physicians. Probert won't be satisfied until he gets into jail again.—*Digest.*

**Factors of Success.** No external advantages can compensate the lack of essential qualifications in the workman himself. Never were these qualifications more important than to-day, personal qualities rooted in personal character. Just as in the old days, success is the product of the tool multiplied by the man. No instrument brings skill for its own use. Skill is a personal quality, developed by the assiduous training of nature's gifts. The outfit of a dental establishment includes the man. Energy, industry, unfailing patience, blended with integrity, fidelity and the affability of a kindly heart are indispensable to real and permanent success.—Dr. Chambers in *Review.*

**Phenol Monochlorid (para).** This is a crystalline substance partially soluble in water, more readily in alkalies. It is a good antiseptic and disinfectant for putrid canals, or as a preservative after removal of pulps. It can be used in a mixture of iodoform and oxide of zinc as a root filling. It also is a useful antiseptic and analgetic ingredient of pulp devitalizing pastes. In concentration it has been used as a treatment in pyorrhoea alveolaris applied by means of a warm platinum wire once or twice each week. It has a somewhat unpleasant odor, which may be subdued by mixing with menthol. A paste may be made with glycerine.—*Merck's Annual.*

**Damage Suits.** A woman in New York recently sued a dentist for $2,000 damages, claiming that because of his improper treatment of a tooth a scar was left on her face. The dentist proved that an abscess had pointed on the outside of her face before she came to him, and the jury returned a verdict in his favor.—A dentist in New York City has been sued for $25,000 damages by a man who alleges that, owing to the dentist's carelessness in extracting a tooth from the mouth of his son, blood-poisoning set in and caused his death.—A man in Memphis, Tenn., has sued a dental parlor in that city for $5,000 damages, alleging that the operator extracted a sound tooth by mistake.—*Digest.*

**Get Acquainted with your Instruments.** If I buy a new tool and it is complicated, the first thing I do is to pull it apart and thereby I enjoy a great deal of pleasure and afford a great deal of pleasure to my friends, and, incidentally, make an extra amount of dealing with the dental depots; but if I have ruined one tool, the next tool will last me three times as long as the first one would have done if I had not pulled the former one apart to know how to tighten it, how to take care of it or repair it, but if I can get it together again, I have as good a tool as ever I had. If I can't, the next time I buy one I know how to take it apart and how to take care of it.—Dr. Hungerford in *Western Journal.*

**Bridges.** We can all learn a lesson by looking at the three bridges which span the Niagara River. One is a suspension, another a cantilever and the third an arch. The engineers who planned them knew exactly what was needed at the various points, also what would best meet the needs, and each bridge is doing its work perfectly. It would be senseless to make them all alike or to substitute one for another. It is just as senseless to say that all bridges in the mouth must be made just the same and upon the same principles. So long as there are differences in the teeth, gums and alveolar processes upon which we operate, so long there must be differences in the structures which we build and the methods we employ. We might just as well plead for uniformity in the shapes of fillings as for the uniformity in bridges.—Dr. Hewitt, in *Digest.*

**Plate Making.** Dentists practicing fifteen years ago extracted hundreds of teeth that we are saving to-day. Operative dentistry has advanced, and whenever we have an advancement of one branch, it is always at the sacrifice of another. It is not because dentists do not want to do prosthetic dentistry so much, but the conservative dentist necessarily must do less prosthetic work. We must be doing things continually to become proficient. It is the same with the student; you must keep him working day in and day out. If one stands at the chair and fills teeth for five or six hours a day, and does little prosthetic work, what is the result? He naturally loses in that branch, and I believe this is one explanation for the conditions we have all over this country to-day. We condemn our advertising dentists for constructing dentures for five or six dollars, but as long as the average reputable dentist does not take any more interest than he does, and gives no better results, we cannot blame people for going to men and paying five dollars for their plates if they get as good results as they can from reputable dentists for ten or fifteen dollars.—*Review.*

**Proprietary Medicines.** Another step in the rapidly growing movement to place the proprietary medicine business on a basis where it must depend upon merit rather than "bunkum." The American Medical Association at its New Orleans meeting in May, resolved:

That newspapers which do not print objectionable medical advertisements are entitled to and should receive the favor and preference of medical men.

That articles on the dangers arising from the use of quack nostrums should be written for such newspapers for publication.

That the Committee on National Legislation be asked to consider the feasibility of the introduction in the next House of Representatives of an interstate measure prohibiting or limiting the sale of poisonous and dangerous patent medicines.

That no medical preparation for internal use, as distinguished from antiseptics, disinfectants, cosmetics and dietetics, advertised as a remedy or cure to the laity, is entitled to the patronage of physicians, nor should such be admitted to the pages of medical journals, nor to the exhibitions of the American Medical Association.

**Illegal Practitioners.** July 9, a dentist at La Junta, Colo., was arrested and fined $100 for practicing dentistry without a license. The fine was suspended on his promising to leave town at once.—We stated last month that the license of a dentist at Omaha had been revoked because of unprofessional conduct. He appealed the case, and the State Board of Health has upheld the action of the Dental Board in revoking the license.—A dentist in New Jersey was arrested some time ago for practicing dentistry without a license. The judge in the lower court found him guilty, as did the Supreme Court, and the case has now been taken to the Court of Errors and Appeals. The man's lawyers contend that because he practiced in the State before the dental law was passed it is not necessary for him now to take out a license.—Characterizing their methods as a "great swindle," a judge in Philadelphia, on July 2, sentenced George C. Courtwright, president of the Alba Dental Co., and Wm. Powell, its manager, to one year and three months' imprisonment, respectively, upon their conviction of conspiracy to defraud. Courtwright was not a graduate of any dental college, and it was proven that students were employed and that untruthful advertising was done. A few such lessons as this would drive the fakirs out of business.—*Digest.*

**Amalgam Filling.** I have found it almost impossible for me to burnish amalgam into a small groove so that no space is unoccupied by the filling material when the amalgam has thoroughly set. Under such treatment I have generally found that there did exist an appearance indicating that such a space had been filled with amalgam containing an excess of mercury, which, after thorough crystallization, had disappeared.

When we learn that each point of our cavity must be reached and filling condensed by direct pressure (and a light blow is always best), just as carefully as we would do with gold, then will we learn, as I have done, to make friends with a good amalgam containing a large percent of silver (60 percent or more). Then we will find that there is not so much shrinkage and expansion to complain of. Nor will we have to record so many failures, as in the past. Let us prepare our cavity with all possible care, omitting nothing in haste; then observe these precautions and not be afraid to get well compensated for it. The complaints against us for getting good rates for our efforts, zealously put, will prove finally our best advertisements.

I have found that a gold filling will usually render better service when the cervical margin is well lined with amalgam or tin. And here, too, thorough condensation is very important. I find that gold will adhere and form a union in such a case, although the amalgam filling be thoroughly crystallized when the gold is.—Dr. Marshall in *Texas Journal.*

